# Naphthalene-Induced Methemoglobinemia With Acute Hemolysis in a Child: A Rare Case of Refractory Hypoxia

**DOI:** 10.7759/cureus.105442

**Published:** 2026-03-18

**Authors:** Rajeeva Mishra, Prativa Gari, Katyayani Rai, Fauzia Zarrin

**Affiliations:** 1 Pediatrics, Rajendra Institute of Medical Sciences, Ranchi, IND

**Keywords:** blood gas analysis, co-oximetry, methemoglobinemia, methylene blue, pulse oximetry, saturation gap

## Abstract

Methemoglobinemia is a rare but potentially life-threatening condition caused by oxidation of hemoglobin iron from the ferrous to ferric state, impairing oxygen delivery to tissues; early recognition is crucial as clinical manifestations may be disproportionate to pulse oximetry readings. We describe a case of a three-year-old female child who presented with acute onset cyanosis and hypoxia unresponsive to supplemental oxygen, despite a normal cardiopulmonary examination and persistently low oxygen saturation on pulse oximetry. Blood gas analysis revealed a discrepancy between low oxygen saturation and oxygen levels, raising suspicion of methemoglobinemia, which was subsequently confirmed by elevated methemoglobin levels. The child was managed with prompt supportive care, including high-flow oxygen therapy, followed by intravenous methylene blue, due to significant symptoms and elevated methemoglobin levels, which resulted in rapid clinical and biochemical improvement. She made a complete recovery without residual complications. This case underscores the importance of considering methemoglobinemia in young children presenting with unexplained hypoxia refractory to oxygen therapy, as timely diagnosis and appropriate treatment can be life-saving.

## Introduction

Methemoglobinemia is a rare but potentially life-threatening disorder caused by oxidation of ferrous iron (Fe²⁺) in hemoglobin to the ferric (Fe³⁺) form, resulting in impaired oxygen transport and tissue hypoxia [1,2]. Under normal physiological conditions, methemoglobin levels are maintained below 1%-2% of total hemoglobin through the action of enzymatic reduction pathways, primarily the cytochrome b5 reductase system. Failure of these mechanisms or increased oxidative stress leads to the accumulation of methemoglobin and subsequent clinical manifestations [3].

In the pediatric population, methemoglobinemia may be either congenital or acquired, with the acquired form being far more common [4]. Acquired methemoglobinemia is most often associated with exposure to oxidizing agents, including nitrates, nitrites, local anesthetics such as benzocaine and prilocaine, dapsone, and various chemicals or household substances [5]. Naphthalene, a commonly available household product in India used for protecting clothes from insects, is a known cause of pediatric poisoning and has rarely been reported as an etiological agent for methemoglobinemia [6]. Naphthalene is metabolized to oxidative metabolites such as naphthoquinone, which oxidize hemoglobin iron from ferrous to the ferric form, producing methemoglobin and causing oxidative damage to erythrocytes, leading to hemolysis.

Clinically, methemoglobinemia presents with cyanosis that is disproportionate to respiratory findings and does not improve with oxygen therapy [7]. Associated symptoms include dyspnea, fatigue, headache, dizziness, altered sensorium, seizures, and hemolysis at higher methemoglobin levels [8]. A characteristic diagnostic feature is the presence of a saturation gap, where pulse oximetry shows low oxygen saturation despite a relatively normal arterial oxygen tension [9].

Management includes immediate cessation of exposure to the offending agent and administration of intravenous methylene blue, which accelerates the enzymatic reduction of methemoglobin via the NADPH-dependent pathway [10]. Early diagnosis and prompt treatment are associated with excellent outcomes. Given the rarity of naphthalene-induced methemoglobinemia in children, this case highlights an unusual etiology and emphasizes the importance of maintaining a high index of suspicion in pediatric poisonings presenting with unexplained hypoxia.

## Case presentation

**Figure 1:**
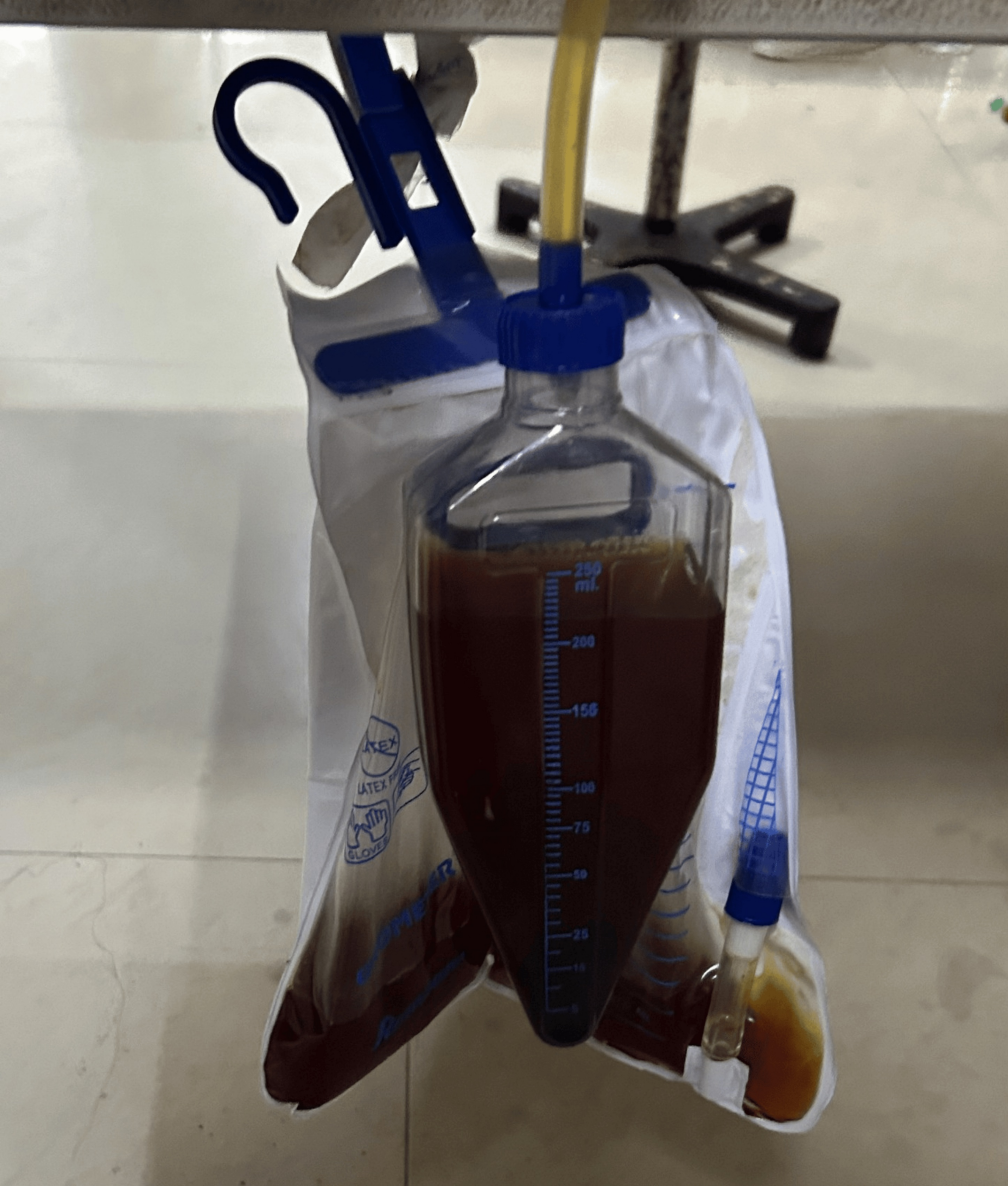
Dark-coloured urine as observed on presentation

A three-year-old female child was brought to the emergency department with complaints of acute onset nausea, vomiting, and passage of dark-colored urine (Figure 1). The symptoms developed a few hours after a definite history of accidental ingestion of naphthalene balls. There was no history of fever, seizures, cough, breathlessness, or prior drug intake.

Physical examination

On examination, the child was conscious and oriented, with a Glasgow Coma Scale (GCS) score of E4V5M6. General examination revealed pallor, while icterus, cyanosis, clubbing, lymphadenopathy, and edema were absent. Vital parameters showed a heart rate of 92/min, respiratory rate of 38/min, and oxygen saturation (SpO₂) of 68% on room air, despite the absence of respiratory distress (chest retractions, nasal flaring, or tachypnea). Systemic examination was normal.

Laboratory studies

The results of laboratory investigations are shown in Tables 1-5.

**Table 1:** Baseline arterial blood gas analysis at admission

Parameter	Result	Reference values
pH	7.341	7.35-7.45
pCO_2 _(mmHg)	38.7	35-45
pO_2_ (mmHg)	39.1	80-100
Bicarbonate (HCO3-) (mmol/L)	20.6	22-26
Base excess (mmol/L)	-4.8	-2 to +2
Oxygen saturation (sO_2_) (%)	51.5	95-100
Oxyhemoglobin (O_2_Hb) (%)	44.5	95-98
Carboxyhemoglobin (COHb) (%)	2.2	<2
Reduced hemoglobin (HHb) (%)	41.8	<5
Methemoglobin (MetHb) (%)	11.4	<1.5
Lactate (mmol/L)	4.12	0.5-2.2
Sodium (mmol/L)	152.2	135-145
Potassium (mmol/L)	4.68	3.5-5.0
Glucose (mg/dL)	129.5	70-110
Hemoglobin (g/dL)	5.77	11-13.5
Hematocrit (%)	23	34-40
Ionized calcium (mmol/L)	1.1	1.12-1.32

**Table 2:** Arterial blood gas analysis after starting treatment

Parameter	Result	Reference values
pH	7.39	7.35-7.45
pCO_2_ (mmHg)	40	35-45
pO_2_ (mmHg)	96	80-100
Bicarbonate (HCO3-) (mmol/L)	23	22-26
Base excess (mmol/L)	-2.73	-2 to +2
Oxygen saturation (sO_2_) (%)	88	95-100
Oxyhemoglobin (O_2_Hb) (%)	78	95-98
Carboxyhemoglobin (COHb) (%)	0.9	<2
Reduced hemoglobin (HHb) (%)	8	<5
Methemoglobin (MetHb) (%)	8	<1.5
Lactate (mmol/L)	3	0.5-2.2
Sodium (mmol/L)	148	135-145
Potassium (mmol/L)	4.4	3.5-5.0
Glucose (mg/dL)	108	70-110
Hemoglobin (g/dL)	6	11-13.5
Hematocrit (%)	21	34-40
Ionized calcium (mmol/L)	1	1.12-1.32

**Table 3:** Post-treatment arterial blood gas analysis

Parameter	Result	Reference values
pH	7.48	7.35-7.45
pCO_2_ (mmHg)	23.1	35-45
pO_2_ (mmHg)	99	80-100
Bicarbonate (HCO3-) (mmol/L)	19.2	22-26
Base excess (mmol/L)	-2	-2 to +2
Oxygen saturation (sO_2_) (%)	99	95-100
Oxyhemoglobin (O_2_Hb) (%)	94.8	95-98
Carboxyhemoglobin (COHb) (%)	1	<2
Reduced hemoglobin (HHb) (%)	0.9	<5
Methemoglobin (MetHb) (%)	3.4	<1.5
Lactate (mmol/L)	2.11	0.5-2.2
Sodium (mmol/L)	143.4	135-145
Potassium (mmol/L)	4.27	3.5-5.0
Glucose (mg/dL)	99.2	70-110
Total hemoglobin (g/dL)	10.43	11-13.5
Hematocrit (%)	33.5	34-40
Ionized calcium (mmol/L)	1.069	1.12-1.32

**Table 4:** Complete blood count MCV: mean corpuscular volume; MCH: mean corpuscular hemoglobin; MCHC: mean corpuscular hemoglobin concentration

Parameter	Result	Reference values
Hemoglobin (g/dL)	5.89	11-13.5
RBC count (x10^6^/µL)	1.9	4.0-5.2
Hematocrit (%)	15.9	34-40
MCV (fL)	83.5	75-87
MCH (pg)	31	24-30
MCHC (g/dL)	36.2	32-36
Platelet count (x10^3^/µL)	458	150-450
Total leukocyte count (x10^3^/µL)	23.4	5.0-15

**Table 5:** Other laboratory investigations

Investigation	Result	Reference values
G6PD level	13.08 U/g of Hb	7-20 U/g Hb
Direct Coombs test	Negative	Negative
Peripheral smear for malaria (thick and thin)	Negative	No parasite seen

Management

Immediate supportive care was initiated, and packed red blood cell transfusion was given in view of severe anemia. Specific therapy was given with 1% methylene blue at a dose of 2 mg/ kg intravenously over 5-10 minutes, and the dose was repeated after one hour due to persistent desaturation. Continuous monitoring of oxygen saturation, vital parameters, and urine color was done.

Outcome

The child showed rapid clinical improvement, with normalization of oxygen saturation and resolution of high-colored urine. Methemoglobin levels decreased significantly following treatment. Hence, the patient had a favourable outcome with timely intervention.

## Discussion

Methemoglobinemia is an uncommon but potentially life-threatening cause of hypoxia in children, resulting from oxidation of hemoglobin iron from the ferrous (Fe²⁺) to ferric (Fe³⁺) state, rendering it incapable of binding oxygen and impairing tissue oxygen delivery [1]. Under physiological conditions, methemoglobin levels are maintained below 1%-2% by enzymatic reduction systems, predominantly the cytochrome b5-NADH reductase pathway, as described in standard pediatric texts [1,4].

Acquired methemoglobinemia is far more common than congenital forms and usually follows exposure to oxidizing agents such as drugs, chemicals, or toxins. In children, accidental ingestion of household substances remains a significant cause, especially in developing countries. Naphthalene, a volatile aromatic hydrocarbon compound widely used in Indian households as an insect repellent and deodorizer, acts as a potent oxidizing agent and has been implicated in hemoglobin oxidation and hemolysis [10].

The presence of high-colored urine and severe anemia in this case suggests concomitant oxidative hemolysis, a known complication of exposure to oxidant toxins, including naphthalene [11]. The index child presented with profound hypoxemia (SpO₂ 68%) without signs of respiratory distress, a classical saturation gap, which is a hallmark feature of methemoglobinemia and should prompt immediate diagnostic consideration [12]. The absence of obvious cyanosis in this patient can be attributed to severe anemia, as cyanosis depends on the absolute concentration of deoxygenated hemoglobin rather than oxygen saturation alone, a concept well described in pediatric physiology.

Blood gas analysis in methemoglobinemia typically reveals a normal or elevated PaO₂ despite low oxygen saturation readings on pulse oximetry, highlighting the limitations of standard pulse oximeters and the importance of co-oximetry for definitive diagnosis [12]. Although direct methemoglobin levels were not documented, the dramatic clinical response to methylene blue strongly supports the diagnosis.

Methylene blue remains the treatment of choice for symptomatic methemoglobinemia or when methemoglobin levels exceed 20%-30%. It acts as an artificial electron carrier, facilitating the NADPH-dependent reduction of methemoglobin back to functional hemoglobin. The recommended dose of 1-2 mg/kg administered intravenously was used in this patient, resulting in rapid improvement in oxygen saturation. Normal G6PD levels allowed safe administration, as methylene blue may worsen hemolysis in G6PD-deficient patients.

Supportive management, including oxygen therapy and packed red blood cell transfusion, was crucial in correcting severe anemia and improving oxygen-carrying capacity. With early recognition and timely intervention, the prognosis of acquired methemoglobinemia in children is excellent, as also observed in previously reported pediatric cases [6].

## Conclusions

Methemoglobinemia is a rare but critical cause of hypoxia in children and should be suspected when oxygen saturation is disproportionately low compared to clinical findings. The acquired form is the most common and frequently occurs due to exposure to oxidizing agents, including household substances such as naphthalene. Preventive strategies, including parental education and safe storage of household toxins, are vital to reduce accidental pediatric exposures.
